# Modern aridity in the Altai-Sayan mountain range derived from multiple millennial proxies

**DOI:** 10.1038/s41598-022-11299-1

**Published:** 2022-05-11

**Authors:** Olga V. Churakova-Sidorova, Vladimir S. Myglan, Marina V. Fonti, Oksana V. Naumova, Alexander V. Kirdyanov, Ivan A. Kalugin, Valery V. Babich, Georgina M. Falster, Eugene A. Vaganov, Rolf T. W. Siegwolf, Matthias Saurer

**Affiliations:** 1 Siberian Federal University, Svobodny pr. 79, 660041 Krasnoyarsk, Russia; 2 Swiss Federal Institute for Forest, Snow and Landscape Research WSL, Zürcherstrasse 111, 8903 Birmensdorf, Switzerland; 3 V.N. Sukachev Institute of Forest SB RAS, Federal Research Center “Krasnoyarsk Science Center SB RAS”, Akademgorodok 50 bld. 28, 660036 Krasnoyarsk, Russia; 4 V.S. Sobolev Institute of Geology and Mineralogy, pr. Akademika Koptyuga 3, 630090 Novosibirsk, Russia; 5 Research School of Earth Sciences, The Australian National University, ACT, Canberra, Australia

**Keywords:** Climate-change ecology, Ecophysiology, Stable isotope analysis, Biogeochemistry, Climate sciences, Ecology, Environmental sciences, Hydrology, Limnology

## Abstract

Temperature and precipitation changes are crucial for larch trees growing at high-elevation sites covered by permafrost in the Altai-Sayan mountain range (ASMR). To contextualize the amplitude of recent climate fluctuations, we have to look into the past by analyzing millennial paleoclimatic archives recording both temperature and precipitation. We developed annually resolved 1500-year tree-ring cellulose chronologies (δ^13^C_cell_, δ^18^O_cell_), and used these new records to reconstruct the variability in local summer precipitation and air temperature. We combined our new local reconstructions with existing paleoclimatic archives available for the Altai. The data show a strong decreasing trend by ca. 49% in regional summer precipitation, along with a regional summer temperature increase towards the twenty-first century, relative to the preceding 1500 years. Modern dry conditions (1966–2016 CE) in the ASMR are the result of simultaneous summer warming and decreased precipitation. Our new reconstructions also demonstrate that climate change in the ASMR is much stronger compared to the global average.

## Introduction

Paleoclimate records provide important constraints on the natural range of air temperature and precipitation patterns from local to global scales, allowing contextualization of modern conditions. This is particularly the case for the Common Era (CE), where high-resolution paleoclimate information is available. Over the past two decades, large-scale data synthesis efforts by the international Past Global Changes 2k network have produced robust estimates of regional to global air temperature patterns^1,2^. However, the water cycle is a complex target compared to surface air and ocean temperature; different climate archives track changes in different aspects of the water cycle in different ways, and variability in the water cycle is highly spatially heterogeneous^3^. For example, annual or summer precipitation amount at any given location on the Earth’s surface is governed not just by atmospheric processes that deliver moisture to the region, but also by topography. Long-term precipitation variability in high-elevation regions is particularly poorly understood^4^.

Precipitation amount and variability, as well as temperature, are crucial for trees that grow in high-elevation regions under severe climate conditions in the permafrost zone. Once such region is the Altai-Sayan mountain range (ASMR) region in inner Asia, which covers 1,065,000 km^2^, and represents vegetation types from the steppe ecotone to taiga forests. The frequency of extreme events has increased in the ASMR over the past decades, affecting forests and humans^5,6^. However, long records of paleoclimate variability are still rare from remote vulnerable forest ecosystems such as the ASMR, making it difficult to determine whether these events are within the range of long-term internal variability, or a likely consequence of anthropogenic climate change.

Air temperature signals can be recorded in various paleoclimatic archives like tree rings^7^, ice cores, and lake sediments^8^, with different temporal and spatial resolutions. High-elevation paleoclimate reconstructions derived from larch tree rings may span centuries and even several millennia, at annual resolution^9–11^. Tree-ring width (TRW) and maximum latewood density (MXD) chronologies obtained from long-living larch trees growing in the ASMR primarily preserve a local summer^12–17^. The ASMR region is particularly valuable for paleoclimate research because of the existence of old trees and their preservation for millennia^9,13^^.^

Air temperature and precipitation changes may also be recorded in stable carbon and oxygen isotopes of plant organic matter^18,19^. Measurements of the stable carbon isotopic composition of tree-ring cellulose (δ^13^C_cell_) covering the past 200 years have demonstrated the potential for building summer precipitation amount reconstructions for the ASMR^20,21^. On the other hand, the stable oxygen isotope composition of tree-ring cellulose (δ^18^O_cell_) has been proven as a useful proxy for temperature variability at high-elevation sites^20,21^. The stable isotopic composition of tree-ring cellulose is therefore a valuable archive of both temperature and precipitation information for the ASMR.

Aside from tree-ring based paleoclimate information, other proxy types available in the ASMR region record information about temperature and precipitation. Glacier ice cores and lake sediments record annual, spring, summer and autumn air temperature changes, although at lower (decadal) resolution^21–23^. The concentration of several elements in lake core sediments (Ca, Ti, Br, and Sr) reflect temperature changes, whilst elements from clastic minerals (Rb/Sr) may reflect precipitation and runoff signals^24^. The ratio of coherent (Co) to incoherent (Inc) scatter from micro-XRF core scanning data have also been interpreted in terms of precipitation variability^25^. Together, these proxy types should provide a comprehensive understanding of regional climatic variability in the ASMR, however the information has not previously been combined in a quantitative reconstruction.

In this study, we provide a comprehensive description of the climatic changes at the ASMR, using a multi-parameter approach to test whether modern climatic changes (1900–2016 CE) are anomalous compared to the preceding millennia. We (i) present new annually resolved 1500-year-long stable carbon and oxygen isotope chronologies from Siberian larch tree-ring cellulose (δ^13^C_cell_ and δ^18^O_cell_), and discuss the controlling influences of climate parameters on stable isotopes; (ii) derive local July precipitation and July air temperature reconstructions based on the δ^13^C_cell_ and δ^18^O_cell_, respectively, and reveal anomalies during the period 516–2016 CE; (iii) develop a regional multi-proxy June–July–August (JJA) air temperature and JJA precipitation reconstructions based on the newly developed δ^13^C_cell_ and δ^18^O_cell_ time series combined with published (Table S1) paleoclimatic records for the Altai region. We then compare ASMR climatic variability in the Recent Period (RP, 1900–2016 CE), and specifically during the past 16 years of the twenty-first century, with the Late Antique Little Ice Age (LALIA, 516–800 CE); the Medieval Warm Period (MWP, 800–1300 CE); and the Little Ice Age (LIA, 1400–1900 CE), in both a regional and a global context.

## Results

### 1500-year stable carbon and oxygen isotopes in larch tree-ring cellulose

**Figure 1:**
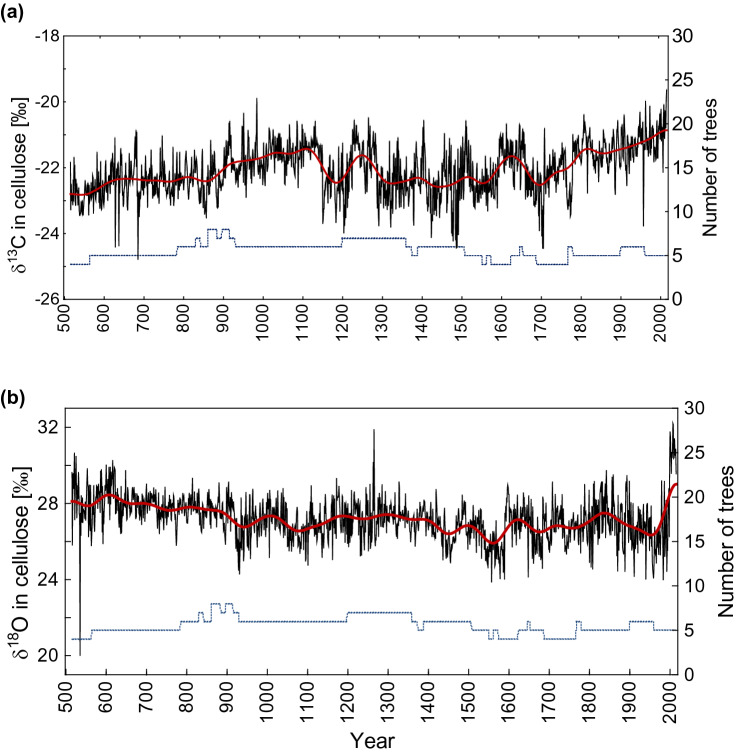
Annually resolved δ^13^C_cell_ (**a**) and δ^18^O _cell_ (**b**) in Siberian larch tree-ring cellulose chronologies for the period from 516 to 2016 CE. Chronologies are smoothed by a 101-year Hamming window to highlight a centennial scale. The dotted and dashed lines indicate the number of trees analysed.

The δ^13^C_cell_ (Fig. 1a, Fig. S2) and δ^18^O_cell_ (Fig. 1b, Fig. S3) records span 516–2016 CE, at annual resolution. The δ^13^C_cell_ timeseries shows mostly increasing trends during the first millennium of the Common Era (516–1120 CE), and similarly at the end of the last millennium (1720–2016 CE). The maximum δ^13^C_cell_ value occurs in 2016 CE (−19.6‰; + 3.2σ), while the minimum occurs in 686 CE (−24.7‰, −3.6σ) relative to the average for the period 516–2016 CE (−22.04‰) (Table S2, Fig. S2). The standard error (SE) for the whole analysed period is 0.02.

The δ^18^O_cell_ timeseries (Fig. 1b, Fig. S3) showed two positive and one negative extreme over the past 1500 years, with the minimum value (19.9‰; −6.3σ), occurring in 536 CE, and maximum values (31.9‰; + 3.8σ and 32.2‰; + 4.4σ), occurring in 1266 and 2008 CE, respectively (Table S2, Fig. S3). The SE for the whole analysed period is 0.03. The δ^18^O_cell_ data has higher standard deviation (SD) (1.15) than δ^13^C_cell_ (0.75).

Less than 1% of values in the δ^18^O_cell_ record are classified as extreme, with the standard deviation ≥  ± 3σ. The δ^13^C_cell_ and δ^18^O_cell_ records are significantly correlated (r = 0.1, p = 0.0001, n = 1500).

### Local climate signals preserved in δ^13^C_cell_ and δ^18^O_cell_ records

We used weather observations from the local Mugur-Aksy weather station (50°N, 90°E, 1850 m asl) (Table S1) to derive quantitative paleoclimatic reconstructions from our δ^13^C_cell_ and δ^18^O_cell_ timeseries. A multiple linear regression analysis revealed significant correlations between δ^13^C_cell_ and July precipitation (r = −0.58; p < 0.0001) (Fig. S4a, Table S4), and δ^18^O_cell_ and July air temperature (r = 0.64; p < 0.0001) (Fig. S4b, Table S5), for the period from 1966 to 2015. These relationships allows us to infer July precipitation from δ^13^C_cell_ (r = 0.49, F = 14.79, df = 1.47, p = 0.0003), and July air temperature from δ^18^O_cell_ (r = 0.61, F = 29.76, df = 1.50, p = 0.0002). The δ^13^C_cell_ is also significantly (but negatively) correlated with the temperature of September in the previous year (r = −0.38; p < 0.05). The δ^18^O_cell_ is significantly positively correlated with the same variable (r = 0.36, p < 0.05), which may indicate that warm and dry climate conditions cause prolongation of vegetation season. The drought index (DRI) averaged over May–July (r = −0.52; p < 0.05) for the period from 1966 to 2016 CE is also significantly correlated with δ^13^C_cell_ (Fig. S4).

### Local and regional precipitation reconstructions

**Figure 2:**
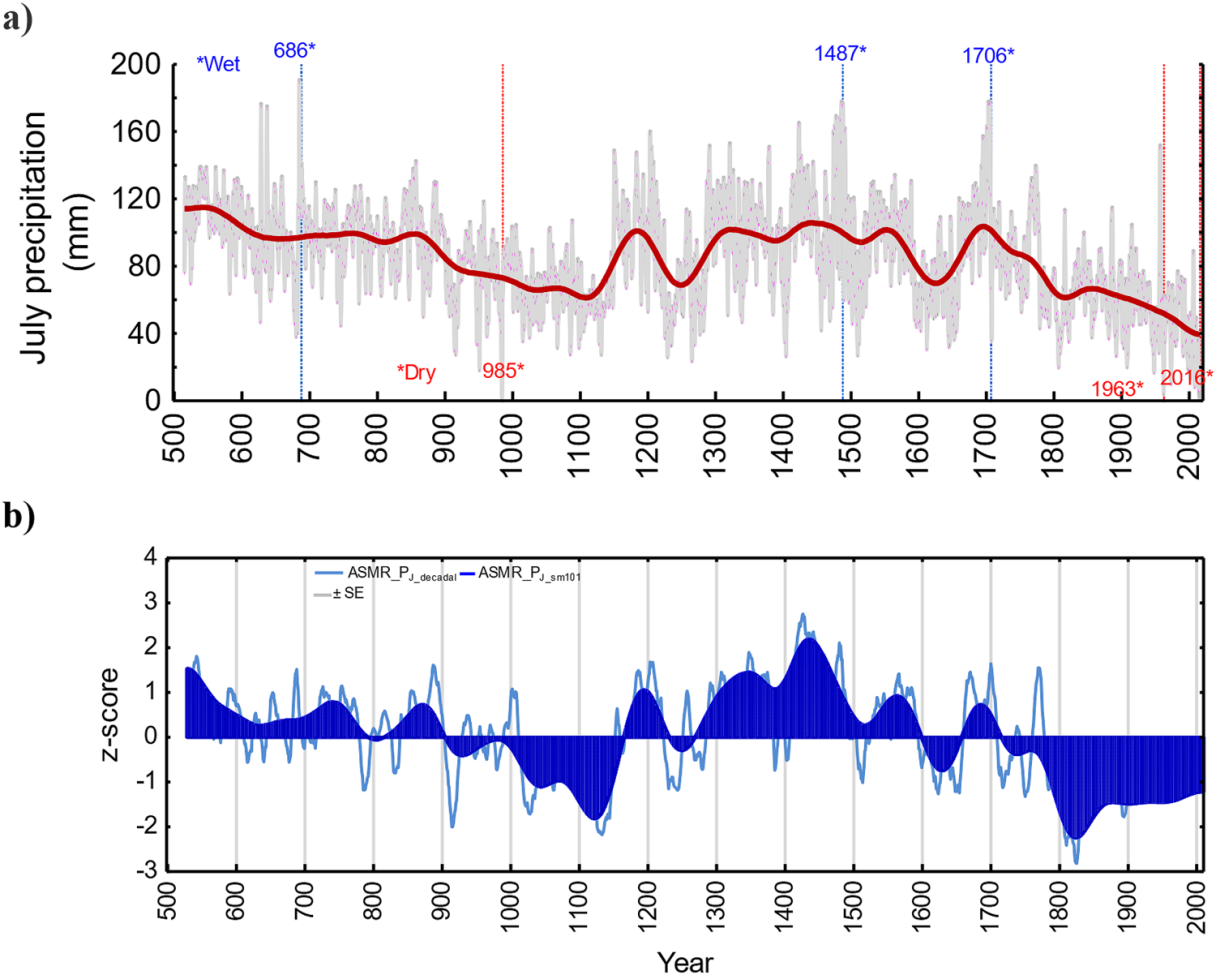
A newly reconstructed annually-resolved July precipitation chronology was derived from the δ^13^C_cell_ for the period from 516 to 2016 CE. Standard errors (SE + and SE−) presented in a grey color (**a**) and a newly developed regional ASMR—July precipitation reconstruction (ASMR-P_J_) based on the combination of δ^13^C_cell,_ Rb/Sr and Co/Inc (Eq. 7) for the period from 529 to 2010 CE (**b**). All chronologies are smoothed by a 101-year Hamming window.

The local July precipitation reconstruction derived from δ^13^C_cell_ only suggests relatively low precipitation (i.e., dry conditions) during the tenth to eleventh, thirteenth, nineteenth, and twenty-first centuries, with relatively high precipitation (i.e., wet conditions) during the seventh, twelfth, fifteenth to sixteenth, eighteenth centuries (Fig. 2a). Our reconstruction also shows pronounced decreasing July precipitation trends from the sixth to twelfth and the eighteenth to twenty-first centuries.

Precipitation extremes (dry < −2.5σ, wet >  + 2.5σ) were calculated across all individual years (Table S6) and for the historical period (Table S7). Recent dry events are superimposed on a pronounced downward precipitation trend (2000–2016 CE) (Fig. 2a, Table S6). The maximum July precipitation occurred in the seventh century, with July precipitation totals reaching double the long-term average (85.3 ± 28.8 mm) of the past 1500 years, with similarly wet years occurring during the LIA (Table S6).

We also calculated a regional multi-proxy ASMR JJA precipitation reconstruction (Table S3), based on our new δ^13^C_cell_ record combined with Co/Inc and Rb/Sr values from the Teletskoe Lake sediment core^26^, where each predictor explains the following amount of variance: δ^13^C_cell_ 43% (Fig. S5a), Co/Inc 33% and Rb/Sr 24% (Fig. S6). The mean correlation coefficient of all possible pairs of proxy datasets is 0.78. Statistical relationships between regional ASMR reconstructed JJA precipitation versus observed JJA precipitation data from the Barnaul weather station over the common period 1930–2009 CE are significant at annual resolution (r = 0.51, r^2^ = 0.25, F = 16.11, df = 1.47, p = 0.0002, standard error of estimate 0.38) as well as for the smoothed by a 10-year average (r = 0.79; p = 0.0001). Correlations remain also stable and significant over calibration and verification periods (Fig. S5a). The first-order difference between the observed JJA precipitation from the Barnaul weather station data and reconstructed JJA precipitation were computed and passed the significance test at p = 0.00019; r = 0.46, F-criteria = 20.8, df = 1.77 (Fig. S5c). Therefore, the two series have good consistency in high-frequency changes and can be proved to be reliable.

In the ASMR JJA regional precipitation reconstruction, strong negative precipitation anomalies occur during the ninth to eleventh, eighteenth to twenty-first centuries (Fig. 2b). Comparable dry intervals occurred during both the MWP and the modern period, albeit with a more pronounced signature in the modern period. The regional reconstruction reveals an unprecedented decreasing trend in ASMR JJA precipitation by almost 49% towards the twenty-first century (1966–2016 CE) compared to the preceding millennium (535–1965 CE).

### Local and regional air temperature reconstructions

**Figure 3:**
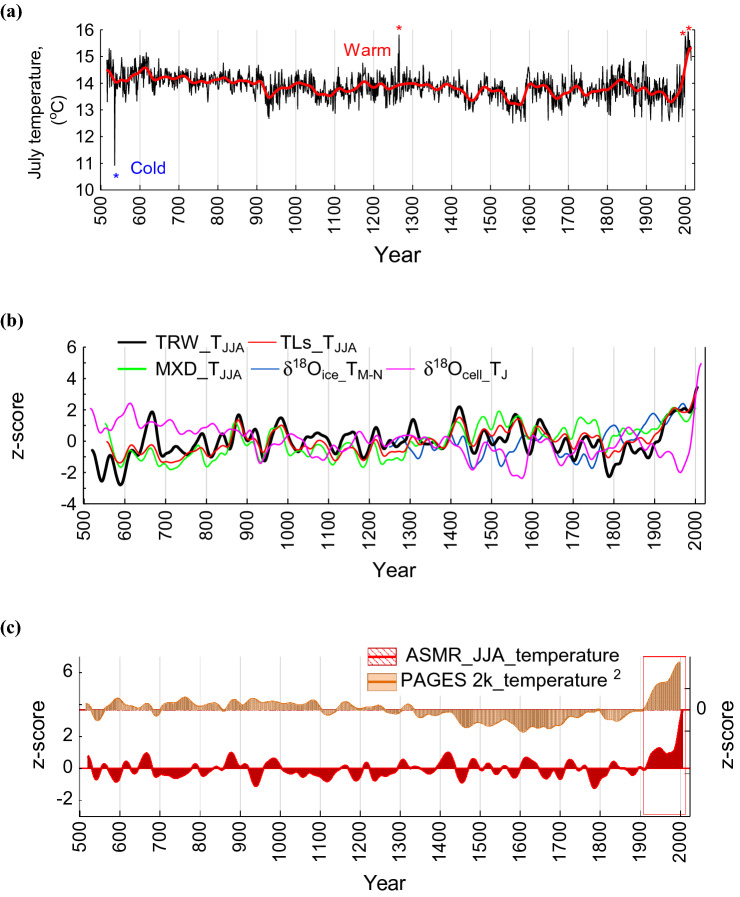
Annually resolved (grey line) and smoothed by a 101-year Hamming window (red line). July air temperature reconstruction are derived from δ^18^O_cell_ for the period from 516 to 2016 CE (this study) (**a**); in comparison with smoothed by a 101-year Hamming window summer June, July, August (JJA) air temperature reconstructions derived from tree-ring width^9,10,13^, maximum latewood density (MXD)^15^. The Teletskoe Lake core sediments (TLs) are derived from the geochemical elements (Ca, Ti, Sr/Br) (this study) and March–November temperature reconstruction inferred from Belukha ice core^27,28^ and the newly developed July temperature reconstruction derived from δ^18^O_cell_ in comparison (**b**); and newly developed Altai-Sayan mountain range (ASMR) JJA-air temperature reconstruction (ASMR-T_JJA_) based on tree-ring parameters (δ^18^O_cell_, TRW, MXD) and geochemical elements from the TLs (Ca, Ti, Sr/Br) in comparison with the PAGES 2k global annual (T_ann_) and smoothed by a 101-year Hamming window (T_sm101_) air temperature reconstruction^2^ (**c**).

The July local air temperature reconstruction derived from δ^18^O_cell_ values reveals one particularly cold year (536 CE), during which July air temperature dropped down to 10.9 °C (− 6 σ) compared to the long-term reconstructed average of 14.9 °C for the period 516–2016 CE (Fig. 3a, Table S6).

Reconstructed July air temperature reached a maximum of + 15.8 °C during the MWP (800–1300 CE), which is comparable with prevailing temperatures during the recent period (Table S7). Despite single-year anomalies in the pre-industrial that are comparable to those in the modern period (Table S6), centennial-scale trends and variability demonstrate that the warming at the beginning of twenty-first century is both more pronounced and more persistent compared to the past 1500 years. The JJA-air temperature reconstructions from other temperature-sensitive proxy records in the region (TLs, MXD and TRW, Fig. 3b)^13,15,16,27^ show high synchronicity in their low-frequency variability over the period 529 to 2007 CE. Similarly, our new local July air temperature reconstruction derived from δ^18^O_cell_ shows similarities with tree-ring and lake sediment proxies when smoothed by a 101-year Hamming window. However, the shorter March-November temperature reconstruction from the Belukha ice core glacier (Fig. 3b), is less similar, and this highlights either spatial heterogeneity in local temperature, or a discrepancy in the climatic signal preserved by these different proxy archives during early first millennia and towards the third one (Fig. 3b).

In the ASMR JJA-air temperature reconstruction based on the multiple proxies, the variability is explained by the geochemical elements of Ca by 8%, Br/Sr by 8%, MXD by 26%, δ^18^O_cell_ by 6%, and TRW by 41%. Statistical relationships between observed JJA air temperature from the Barnaul weather station data and reconstructed ASMR JJA regional air temperature are significant at annual resolution (r = 0.56, r^2^ = 0.31, F-criteria = 17.85, df = 1.40, p = 0.0003, standard error of estimate 0.71) and for the smoothed (by a 10-year window) reconstruction during the period for which we have observations i.e., 1838–2006 CE (r = 0.73, p = 0.0001, Fig. S5b). For both calibration (1970–2009, r = 0.75, p = 0.0001) and verification (1930–1969, r = 0.79, p = 0.0001) periods (Figure S5b) the correlations are significant with the standard error for reconstructed data (SE ± 0.41). The first-order difference between the observed JJA air temperature from the Barnaul weather station data and reconstructed JJA air temperature were computed and passed the significance test at p = 0.00000; r = 0.51, F-criteria = 59.28, df = 1.16 (Fig. S5d). Therefore, the two series have good consistency in high-frequency changes and can be proved to be reliable.

The LIA (1400–1900 CE) is strongly marked in the ASMR region in the local δ^18^O summer temperature regional reconstruction (Fig. 3a,b). The extremely cold summer of the year of 536 CE is captured by annually resolved δ^18^O_cell,_ showing the lowest value over the past 1500 years in comparison with other stratospheric volcanic eruptions 1259 CE, 1641 CE, 1815 CE, 1991 CE. This signal is also represented in the regional JJA temperature reconstruction (Fig. 3c), however, with a less pronounced reduction of summer air temperature.

According to our δ^18^O_cell_-based local July air temperature reconstruction, July 2008 CE was the warmest month in the reconstructed period (+ 15.9 °C), with similarly high temperatures occurring only during the MWP. Our new multi-proxy regional JJA temperature reconstruction reveals unprecedented air temperature increase towards the twenty-first century. This can be compared with the observed modern global temperature increase relative to the last millennia, which has a similar shape, but less strong increase^2^ (Fig. 3c).

Our new local and regional summer temperature and precipitation reconstructions demonstrate that modern dryness in the ASMR is unprecedented in the context of the past 1500 years. This leads to higher risk of forest fires, evidence for which is recorded by the ASMR paleo archives^6,26–28^.

Although some single-year temperature extremes (Table S6) were observed in the past of a similar magnitude to the modern period, the frequency of these events was not as high as that of recent drought. Recent aridity was reported for eastern China based on instrumental observations and reconstructed rainfall^29^, which is in line with our finding for the ASMR region. High drought frequency is also observed in Mongolia^30^ and captured in Palmer Drought Severity Index (PDSI) reconstruction for Europe^31^ and in the Swiss Alps^32^, again suggesting that the drought conditions over the recent decades in many parts of Eurasia are unusual in a long-term context.

The September air temperature of the previous hydrological year is negatively correlated with δ^13^C_cell_, but positively correlated with δ^18^O_cell_. This relationship is in line with observations during unusually warm and dry conditions during recent decades; such conditions may limit carbon assimilation and therefore late-season conditions in the previous year are important for accumulating carbon as starch for storage^19^. A hydrologic link between δ^18^O in precipitation and temperature (Figure S4) is well known^18^ and therefore can also be expected for tree-ring δ^18^O in cellulose as it was reported for Chinese monsoon region^29^. For δ^13^C_cell_, an influence of precipitation is expected via the stomatal conductance, which is known to respond to dry conditions^19^.

In the multi-proxy regional ASMR summer precipitation reconstruction, the dry LIA period is most pronounced during 1820–1870 CE. This is in line with paleo- precipitation reconstructions from European sites, e.g., high-elevated sites at the Swiss Alps, where LIA was recorded by the stable carbon isotope proxy indicating a drought^32^. However, LIA is heterogeneously recorded in hydroclimatic conditions world-wide^33^ and specifically for Asian regions, where monsoons affect climate patterns significantly^29,34,35^.

Our local July temperature reconstruction indicates that the coldest year of the past 1500 years occurred in 536 CE (−6σ), and that this summer has no analogues over this past period (Fig. 3a). This corresponds with a major “unknown” volcanic eruption that occurred most likely in 536 CE in the tropics^36^, which is confirmed not only regionally^10,11,17^ but also globally^8^ and possibly resulted in the Northern Hemisphere LALIA^10^.

Our ASMR regional JJA-temperature reconstruction indicates that the average temperature during the recent period (2000–2016 CE) is 2 °C higher than the average for the preceding 1490 years, which is in line with the global-mean temperature reconstruction^1^. Maximum temperature anomalies showed up to 4 °C summer air temperature increase for the single years during the period 2000–2016 CE. Moreover, the ASMR JJA-temperature reconstruction shows similar patterns over the recent decades as the PAGES 2k global mean surface temperature (GMST) reconstruction^2^, emphasizing an unusual recent warming trend towards 2000s. However, the MWP and LIA in the regional ASMR JJA-temperature reconstruction show differences in magnitude and duration compared with the same intervals in the PAGES 2K GMST reconstruction^2^_._. Causes of these differences may be related to external factors, e.g., volcanic eruptions, permafrost and solar variability, and atmospheric patterns^37^.

Specifically, some of the differences between the GMST reconstruction and our regional ASMR JJA air temperature reconstruction can be explained by seasonal effect of different proxies, similar as it was shown by PAGES2k Consortium^2^. The results make it clear that some paleoclimatic archives are sensitive to July, or averaged June–July air temperature, but not or less sensitive to annual air temperature. Despite that, the majority of tree-ring records included in the PAGES 2K global air temperature reconstruction^2^ are from the Northern Hemisphere, there are some proxies with the low spatial resolution, which are included from both Northern and Southern Hemisphere or proxies, which cannot be precisely calibrated in time. It is well known, that the uncertainties for most composites are due to the paucity of records (combination of corals, marine sediments), which show a cooling trend through most the Common Era^2^. The PAGES 2K Consortium^2^ explained this by the low resolution of marine sediment records and local oceanographic factors over the past millennium, as well as the process of bioturbation of the sediment archive. These factors may suppress the ‘true’ signature of climatic changes occurring over years and decades^38^, including the most recent warming.

Simultaneous temperature increase and moisture deficit enhance the drought stress of trees to recent climate change, which is expressed by an increased δ^18^O_cell_ signal towards the twenty-first century. The amplifying effect of vapor pressure deficit on drought stress of trees has been shown globally for many species and sites^39–42^.

Discrepancies between high- and low-temporal resolution proxies are unsurprising and can be explained by different integration times, different driving factors and by recording different seasonal variability (at the beginning of the season early June–until late middle/end of August) or mixed temperature and moisture signals (e.g., thawed permafrost water). Specifically, variability in δ^18^O_cell_ does not only represent local temperature variability, but also information about the VPD and evaporation as hydrological changes^40,43^.

The main discrepancy in our local δ^18^O_cell_—derived July temperature reconstruction, compared with earlier published JJA temperature reconstructions inferred from MXD^10^ and TRW^12^ only, is that it shows a significant temperature increase only over the past few decades. The onset of the recent temperature increase was observed earlier in the TRW and the MXD proxies; this may be because the TRW and MXD proxies contain information about June temperatures, whereas δ^18^O_cell_ is most strongly correlated with July temperature^20^. Furthermore, VPD increases as temperature increases, which is reflected in the δ^18^O_cell_ values as well. Enhanced evapotranspiration and permafrost thaw depth under elevated CO_2_ and temperature increase can impact δ^18^O_cell_ and, therefore, can explain an offset in response to temperature changes towards the third millennia. In contrast, for TRW and MXD summer temperature alone is the dominant driver. The δ^18^O mainly captured the July air temperature signal modulated by drastic permafrost degradation over the recent decades. Information about permafrost thaw soil depth is recorded in the δ^18^O in tree-ring cellulose only in contrast to other tree-ring proxies, which mainly record only air temperature signal (TRW–JJ, and MXD–JA). These differences in seasonal window can average the summer temperature signal, dampen uncertainties and therefore has advantages over single-parameter reconstructions. Offsets in temperature and precipitation reconstructions between individual chronologies can be explained by the use of different weather stations for the calibration period; one weather station each is at low- (180 m a.s.l.) and high-elevation (1850 m a.s.l.). Therefore, using a combination of tree-ring parameters and other paleoclimatic archives provides valuable information about paleo-temperature change, that cannot be obtained from single-proxy reconstructions alone.

## Conclusion

Our study demonstrates the advantage of a multi-parameter records (tree-ring width, latewood density, δ^13^C and δ^18^O in tree-ring cellulose) obtained from the same trees and study site as well as a multi-proxy approach combining tree-ring and lake core sediment proxies from one region. This provided more robust summer air temperature and precipitation reconstructions than would be possible from individual proxies. On one hand, climate reconstructions derived from a single proxy can be a subject to limitations by seasonality and temporal resolution, but on the other hand, can provide site-specific and proxy-specific features, which are reduced in combined generalized chronologies. However, to get robust climate reconstructions we suggest using generalized chronologies.

We showed that a simultaneous summer temperature increase and precipitation decrease occurring during recent decades leading to aridity in the Altai-Sayan mountain range region, which may result in extensive tree declines due to limited water access for Siberian larch trees. Reconstructed surface air temperature and precipitation reveal single years of drought periods during the MWP that can be considered as close to what we are experiencing in the modern period. However, multiple paleoclimatic archives demonstrate that the frequencies of drought anomalies and fluctuation rate of the modern temperature increase and precipitation decrease are unique for the ASMR region for the past 1500 years.

## Materials and methods

### Study site and climate

The study site is located in the Altai-Sayan mountain range (49° N 89° E) (Fig. S1a, Table S1). The climate is continental^44,45^ with an average annual temperature of –2.3 °C. The daily summer temperature ranges from + 9 to + 17 °C at the Mugur-Aksy mountain weather station. Overnight frosts with possible snowfall can occur in July and snow may persist until the end of the month in creek valleys^46^. The rocky screes and sandy sediments are covered by permafrost. The site is characterized by low amounts of precipitation (142.6 mm/year), most of which falls from April until October. The precipitation amount increases with increasing altitudes and the heterogeneous distribution of precipitation patterns is explained by the local orography.

### Sampling of tree cores and stem discs

Siberian larch (*Larix sibirica* Ledeb.) is the main species in the study area on the northern and northeastern slopes of the Mongun Taiga mountain ridge in the western part of the Tuva Republic, Russia (Fig. S1b,c). Tree cores were collected from living trees and stem disc samples were collected from the remaining trunks of dead trees that are well preserved on the permafrost surface at the upper tree line (2300 m a.s.l.). Sampling was performed during several expeditions from 2008 to 2016. Wherever possible, wood collection preference was given to sparse timber stands and isolated growing trees to reduce possible impacts of the presence of other trees on the climatic signal^47^.

### Laboratory analyses

For the analysis of δ^13^C_cell_ and δ^18^O_cell_, we selected 34 discs from dead larch trees, and 8 increment cores from living larch trees (n = 42 trees in total) with an average age of 350 years. Young trees (< 100 years) as well as extremely old trees > 1000 years were excluded from the analysis. In case of narrow rings (< 0.3 mm) up to eight sub-samples from the same wood disc were used to get the required amount of material for the stable isotope analyses. Due to age-related growth trend affecting the tree-ring isotope ratios, the earliest 30 years of the tree-ring series were excluded from the analyses^48^. Traditional measures for assessing the quality of the constructed stable isotope chronologies were used: standard deviation (SD), sample replication for the measurements and standards (Merck reference material), and analysis of samples from each tree individually for the recent period.

Annual tree rings were split manually with a scalpel under a binocular microscope (Leica, Germany) and each ring was enclosed into an individual filter bag. The cellulose extraction was performed according to the laboratory protocol described by Boettger et al.^49^.

The isotopic values are expressed in the conventional delta notation (δ) in (‰) relative to the international standards (Eq. 1):

1 $${\updelta}_{{{\text{sample}}}} = \left( {{\text{R}}_{{{\text{sample}}}} /{\text{R}}_{{{\text{standard}}}} {-}{ 1}} \right) \cdot {1}000$$

where R_sample_ is the ratio of ^13^C/^12^C or ^18^O/^16^O for the sample and R_standard_ is the ratio either of ^13^C/^12^C in the Vienna Pee Dee Belemnite (VPDB) for carbon, or for ^18^O/^16^O in the Vienna Standard Mean Ocean Water (VSMOW) for oxygen.

A good precision (± 0.1‰ for δ^13^C and ± 0.3‰ for δ^18^O) is based on a large number of measurements of the standard material and quality control (n = 99).

As the pyrolysis method via PYRO cube involves a small contribution of carbon from the reactor filling to the measuring gas, the raw δ^13^C measurements were corrected as proposed by Woodley et al.^50^ and further modified by Weigt et al.^51^:

2 $${\updelta }^{{{13}}} {\text{C}}_{{{\text{corrected}}}} = { 1}.{{1142\updelta }}^{{{13}}} {\text{C}}_{{{\text{raw}}}} + { 1}.{45}$$

The δ^13^C_cell_ were corrected for the Suess effect (decline of the ^13^C/^12^C ratio of atmospheric CO_2_) using δ^13^C values of atmospheric CO_2_ obtained from historical data^52^ for the period 1800 to 2016 CE, the South Pole ice core and data from Mauna Loa Observatory, Hawaii related for the recent data http://www.esrl.noaa.gov/gmd/, https://scrippsco2.ucsd.edu/data/atmospheric_co2/mlo.html. This correction is necessary because the emission from fossil fuel combustion and biomass burning have both resulted in decreasing δ^13^C of atmospheric CO_2_ (Fig. S2).

### Development of 1500-year stable isotope chronologies

The δ^13^C_cell_ and δ^18^O_cell_ chronologies cover the period from 516 to 2016 CE, at annual resolution (Fig. 1, S2, S3, Table S1). Where multiple samples overlap chronologically, each annual ring from five trees together was pooled for each year over the period from 516 to 1770 CE (Table S1). Every 10th year, samples from the five trees were individually analysed to check variation coherence among the samples (Fig. S3a).

Samples from the living trees were analysed individually for the δ^13^C_cell_ (Fig. S2a) for the period 1790–2016 CE and then corrected according Francey et al.^52^ for the δ^13^C atmospheric CO_2_ for the period 1801–2016 CE (Fig. S2b), and are also well correlated (r > 0.8; p < 0.01), with an Express Population Signal^53^ (EPS) > 0.9. Therefore, we averaged the δ^13^C_cell_ time series similarly.

The δ^18^O_cell_ for individual trees were measured additionally for the specific time periods (Fig. S3b), with sufficient cellulose material for checking the variability coherence among individual trees. The δ^18^O_cell_ series are significantly correlated (r > 0.5; p < 0.01) among individual trees for the period 2000–2016 CE (Fig. S3b), and we, therefore, averaged the individual records into a single time series.

We used subsamples from the same trees previously used for the construction of millennial TRW^9,10^ and MXD^15^ records, as well as stable isotope chronologies for the short time periods^11,20,21^ (Table S1).

### Statistical analyses

Trends were calculated as the slope of the linear regression from all records. We only discuss regression slopes with a significant (p < 0.05) trend. Statistical characteristics (R—correlation coefficient, R^2^—determination coefficient, F-criteria—Fischer criteria, DW—Durbin-Watson statistics, К_s_—coefficient of synchronicity, CE—covariance error, RE—reduction error) for calibration and verification periods^7,53,54^ between weather station data and paleoclimatic archives were calculated. To characterize long-term centennial trends, we applied a 101-year window by Hamming smoothing^55^. The first-order difference—the change in value from one point in the observed (obs) and reconstructed (rec) time series to the next point as x_t_-x_t-1_ was computed and correlation coefficients were calculated to prove consistency and reliability of the climate reconstructions.

### Quantitative reconstructions derived from new δ^13^C_cell_ and δ^18^O_cell_

We applied multiple regression analysis between δ^13^C_cell_ and δ^18^O_cell_ and air temperature, precipitation, vapor pressure deficit, and sunshine duration from the Mugur-Aksy weather station (1966–2016 CE) (Table S1, Fig. S4a,b) to determine a driving factor impacting stable isotope variation in tree-ring cellulose. To determine the impact of drought on δ^13^C_cell_ and δ^18^O_cell_, we also compared the time series with a drought index (DRI)^56^.

To reconstruct climate back in time we applied regression models, where climatic parameters were the dependent variables, while δ^13^C_cell_ or δ^18^O_cell_ values were independent (Table S3).

After determining that δ^13^C_cell_ is most closely correlated with July precipitation, and δ^18^O_cell_ is most closely correlated with July temperature, we reconstructed the variability over the past 1500 years. Liner regression models were applied between δ^13^C_cell_ and July precipitation (P_July_) data from Mugur-Aksy weather station (calibration period 1966–2015 CE), and between δ^18^O_cell_ and July temperature (T_July_) (Table S3).

### Regional July precipitation reconstruction based on δ^13^C_cell_ combined with Co/Inc and Rb/Sr from Teletskoe Lake core sediments (TLs)

New δ^13^C_cell_ data (this study) were correlated with the ratio of geochemical elements (Rb/Sr) of the TLs, which were previously demonstrated to record precipitation variability, at decadal resolution^26^. Additionally, the ratio of coherent to incoherent scatter (Co/Inc) was used, which was also interpreted as reflecting precipitation variability, via changes in organic matter concentration along the core^25^.

July precipitation from the gridded CRU TS 4.05 land data^56^ for the region 53° N 82° E (Table S1) was used as the reconstruction target, with a calibration period of available data from 1930 to 2009 CE.

A multiple regression equation as a function of δ^13^C_cell_ and ratios of Co/Inc and Rb/Sr in TLs for the common period (529–2010 CE) was calculated (Table S3).

### Regional summer air temperature reconstruction based on δ^18^O_cell,_ TRW, MXD and elemental concentrations (Ca, Ti, Br/Sr) in the Teletskoe Lake core sediments (TLs)

Climatological analysis between the elemental composition of the TLs and climatic parameters was conducted using gridded June–July–August (JJA) air temperature obtained from the KNMI portal https://www.knmi.nl/home (Table S1). The geochemical elements Ca, Ti, Br/Sr were selected for inclusion in the regression model based on the significant correlation of averaged data of these elements with JJA- air temperature (r = 0.53; p < 0.0001).

Previously published TRW^9,10^ and MXD^15^ chronologies showed significant correlation with JJA air temperature (Table S1). Given each of these variables (Ca, Ti, and Br/Sr in TLs, TRW, MXD and our newly developed δ^18^O_cell_) for all of them it has been demonstrated to act as local summer air temperature proxies, using a linear regression model (Table S3). We combined them in a single regional summer air temperature reconstruction (ASMR-JJA). The δ^18^O from the Belukha glacier ice core is also significantly correlated with air temperature (March–November)^28^, however the record spans a considerable shorter time period compared to the other datasets (from 1250 CE), and therefore we excluded it from the ASMR-JJA air temperature reconstruction. Instead, we later used this data for comparison with other proxies.

### Comparison with global scale summer temperature and moisture reconstructions

To place the regional temperature change into a global context, we compared our new ASMR-JJA temperature reconstruction with the Past Global Changes (PAGES 2k) global mean surface temperature reconstruction, which is based on temperature sensitive proxies over the globe^2^. To evaluate our regional precipitation reconstruction in a global context, we used mainly PAGES 2k hydroclimate reconstruction records, including ground ice, speleothems, corals, and marine sediments^3,57^.

### Ethics declarations

Sampling was performed in accordance with relevant institutional guidelines and regulations.

## Supplementary Information

Supplementary Information.

## Data Availability

New data presented in this paper is openly available under CC-BY 4.0 license 10.5281/zenodo.6467290. The MXD raw dataset is available on https://www.ncdc.noaa.gov/paleo/study/18875. The δ^18^O in Belukha ice core glacier water (δ^18^O _ice_) was downloaded from the NOAA repository https://www.ncei.noaa.gov/access/paleo-search/study/12885^28^. PAGES2k temperature data^2^ were obtained from the “figshare”. Mugur-Aksy weather station data used for the calibration period with the stable isotopes in tree rings are available at http://meteo.ru/it/178-aisori (Table S1). Gridded 0.25° × 0.25° (p < 0.01) KNMI temperature, CRU TS4.05^56^ and precipitation data were used from the https://climexp.knmi.nl/getstations.cgi (Table S1).
