# Supplementary material for: Modern aridity in the Altai-Sayan mountain range derived from multiple millennial proxies
Source: Sci Rep. 2022 May 11;12:7752. doi: 10.1038/s41598-022-11299-1 (PMC9095625; doi:10.1038/s41598-022-11299-1)
Supplement: Supplementary file 1 — Supplementary Information. [file 41598_2022_11299_MOESM1_ESM.docx]

**Supplementary Information**

**Modern aridity in the Altai-Sayan Mountain Range** **derived from the multiple millennial proxies**

Olga V. Churakova (Sidorova)^1,2^*, Vladimir S. Myglan^1^, Marina V. Fonti^1,2^, Oksana V. Naumova^1^, Alexander V. Kirdyanov^1,3^, Ivan A. Kalugin^4^, Valery V. Babich^4^, Georgina Falster^5^, Eugene A. Vaganov^1,3^, Rolf T.W. Siegwolf^2^, and Matthias Saurer^2^

^1^Siberian Federal University, Svobodny pr. 79, 660041 Krasnoyarsk, Russia

^2^Swiss Federal Institute for Forest, Snow and Landscape Research WSL, Zürcherstrasse 111 CH-8903 Birmensdorf, Switzerland

^3^V.N.Sukachev Institute of Forest SB RAS, Federal Research Center “Krasnoyarsk Science Center SB RAS”, Akademgorodok 50 bld.28, 660036 Krasnoyarsk, Russia

^4^V.S. Sobolev Institute of Geology and Mineralogy, pr. Akademika Koptyuga 3, 630090 Novosibirsk, Russia

^5^Department of Earth and Planetary Sciences, Washington University in St. Louis, MO 63130 Missouri, USA

Corresponding author: Olga V. Churakova (Sidorova) ([ochurakova@sfu-kras.ru](mailto:ochurakova@sfu-kras.ru))

**
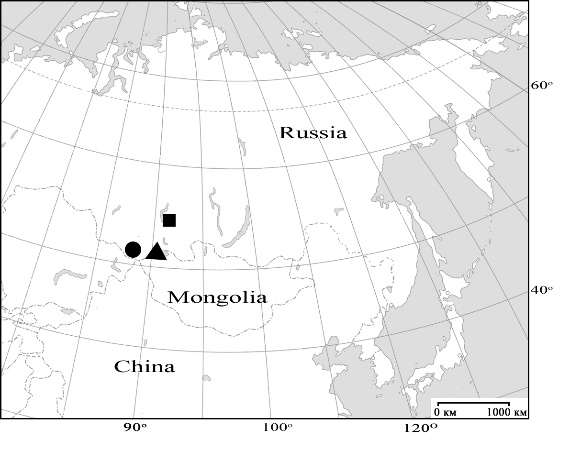
a) b)**

**
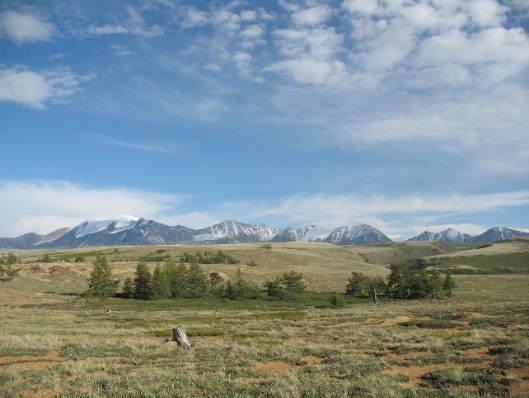
**

**c)**

**
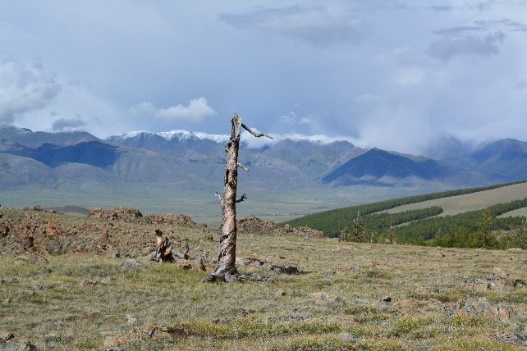
**

**Figure S1.** A multi-proxy study site in the Altai-Sayan Mountain Range (ASMR) (**a**) with location of newly developed millennial carbon (δ^13^C_cell_) and oxygen (δ^18^O_cell_) isotopes (this study), tree-ring width (TRW) ^9, 10, 13^, maximum latewood density (MXD) ^11,15^, (<https://www.ncdc.noaa.gov/paleo/study/18875> (**triangle**), geochemical elements of the Teletskoe Lake core sediments (TLs) ^24,26^ (**square**), and δ^18^O in Belukha ice core glacier (δ^18^O_ice_) ^27,28^; https://data.noaa.gov/dataset/dataset/noaa-wds-paleoclimatology-altai-siberia-750-year-ice-core-d18o-and-temperature-reconstruction) (**circle**) chronologies for regional multi-proxy precipitation and air temperature reconstructions were used. Photos of living (**b**) and stems of the dead (**c**) Siberian larch (*Larix sibirica* Ledeb.) trees archived at the Siberian dendrochronological laboratory, Siberian Federal University, Russian Federation.

1. **b)**

**Figure S2.** Raw **(a)** and corrected according δ^13^C atmospheric CO_2_ **(b)** stable δ^13^C in tree-ring cellulose (δ^13^C_cell_) of the individual trees for the period 1780-2016 CE. Corrected according δ^13^C atmospheric CO_2_ δ^13^C_cell_ values among individual trees showed significant correlations (r=0.6-0.7; *p<0.01),* respectively. The standard deviation (SD) among the δ^13^C_cell_ of individual trees varied from 0.7 to 0.9, average δ^13^C_cell_ values ranged from -19.9‰ to -22.6‰, with the offset from 0.2 to 2.7‰.

**a) b)**

**Figure S3.** The δ^18^O in tree-ring cellulose (δ^18^O_cell_) in individual trees over the past 1500 years (**a**). The δ^18^O_cell_ values among individual trees for the period from 2000 to 2016 CE (**b**) showed significant correlation (r=0.5-0.8; *p<0.01*)*,* respectively. The standard deviation (SD) among of individual oxygen isotope series varies from 0.8 to 1.4. Averaged δ^18^O_cell_ over the period from 2000 to 2016 CE ranged from 30.03 to 31.8 ‰.

**a) b)**

**Figure S4.** Person correlation coefficients calculated between monthly weather station data from Mugur-Aksy for the common period for all parameters (1966 - 2016 CE) and stable isotope chronologies for δ^13^C_cell_ (**a**) and δ^18^O_cell_ (**b**). Temperature (T), vapor pressure deficit (VPD), sunshine duration (Sun), precipitation (P) and drought index (DRI) as an average of May-June-July (DRI_567_). Months represented by numbers from January (Month 1) to December (Month 12). T78 shows averaged July-August air temperature. Dotted red lines show level of significance at *p < 0.05*.

**a) b)**

**c) d)**

**Figure S5.** A multi-parameter Altai-Sayan Mountain Range (ASMR) a 10-year smoothed (**a**) June-July-August (JJA) precipitation (P) reconstruction based on δ^13^C_cell_ and the Teletskoe Lake core sediments versus observed Mugur-Aksy local precipitation data for the periods: 1930-2009 CE (r = 0.78, p= 0.0001); 1930-1969 (r=0.79, p=0.0001); 1970-2009 (r=0.75, p = 0.0001), and (**b**) June-July-August (JJA) air temperature (T) reconstruction based on tree-ring width, latewood density, δ^18^O_cell_ versus averaged observed JJA Barnaul weather station data for the periods: 1838-2006 CE (r=0.73, p = 0.0001); 1838-1922 (r=0.69; p = 0.0001); 1922-2006 (r= 0.54, p = 0.0001). Confidential level (Cl) for the reconstructed JJA temperature and JJA precipitation are represented by ± upper and low levels of standard error (SE) at 95%, which are marked by dotted red lines.

The first-order differences between the observed JJA precipitation from the Barnaul weather station data and reconstructed JJA precipitation were computed and passed the significance test at p = 0.00019; r = 0.46 (**c**). The first-order differences between the observed JJA air temperature from the Barnaul weather station data and reconstructed JJA air temperature were computed and passed the significance test at p = 0.00000; r = 0.51 (**d**).

**Figure S6.** Geochemical elements (Rb/Sr) from the Teletskoe Lake core sediments (TLs) and coherent and incoherent (Co/Inc) scattering data along sediment core in comparison.

**Table S1.** Paleoclimatic archives of the Altai-Sayan Mountain Range region (ASMR).

| **Proxy** | **Temporal resolution** | **Coordinates**  **m a.s.l.** | **Available data**  **Period, CE** | **Weather station** | **Available climate reconstructions** | **References** | **New reconstruction**  **Period, CE** |
| --- | --- | --- | --- | --- | --- | --- | --- |
| δ^13^C_cell_  δ^18^O_cell_ | Annual | 50° N, 89° E  2300 m a.s.l. | 560-899, 1151-1225, 1287-1589, 1661-1800, 2000-2016 | Mugur Aksy  50° N, 90° E,  1850 m a.s.l.  (1966-2015) | NA | This study (new data) | 516-2016  July air temperature  July precipitation |
|  |  |  | 1770-2007 |  | NA | [11, 20, 21] |  |
|  |  |  | 516-580, 890-1160, 1220-1290, 1580-1670 |  | NA |  |  |
| Tree-ring width (TRW) | Annual | 50° N, 89° E  2300 m a.s.l. | 516-2016 | Different gridded products | June-July-August air temperature | [9, 10] | July air temperature  556-2007 |
| Maximum latewood density (MXD) | Annual | 50° N, 89° E  2300 m asl | 556-2007 | CRUTEM 4v network (30-90°N) | June-July-August air temperature | https://www.ncdc.noaa.gov/paleo/study/18875; [15] |  |
| Teletskoe Lake sediments core  (TLs) | Annual, decadal | 51° N, 87° E  434 m a.s.l. | 450-2000 | Barnaul  1930-2010  180 m a.s.l. | Annual precipitation | [22,24] | July precipitation  529-2010  July air temperature |
|  |  |  | 450-2000 | Barnaul  1840-2006  180 m a.s.l. | Annual air temperatures |  | 529-2006 |
|  |  |  |  |  |  |  |  |
| δ^18^O in Belukha ice core glacier  (δ^18^O_ice_) | Age depth model, decadal  (10-yr average) | 406  49° N, 86° E  4062 m a.s.l. | 1255-1975 | Barnaul  1840-2000  180 m a.s.l. | March-November air temperature | <https://data.noaa.gov/dataset/dataset/noaa-wds-paleoclimatology-altai-siberia-750-year-ice-core-d18o-and-temperature-reconstruction>  [27, 28] | NA |

Note: NA – not available

**Table S2.** Mean, minimal (min), maximal (max) and standard deviation (SD) for δ^18^O and δ^13^C in tree-ring cellulose (δ^18^O_cell_ and δ^13^C_cell_) from Altai-Sayan Mountain Range during centennial periods and over the whole studied period 516-2016 CE.

| **Period** | **δ^18^O_cell_** | | | | | | **δ^13^C_cell_** | | | | | |
| --- | --- | --- | --- | --- | --- | --- | --- | --- | --- | --- | --- | --- |
|  | Mean | Min | Max | SD | 25th% | 75th% | Mean | Min | Max | SD | 25th% | 75th% |
| 516-615 | 28.13 | 19.99 | 30.66 | 1.36 | 27.43 | 28.98 | -22.72 | -23.42 | -21.50 | 0.42 | -22.99 | -22.47 |
| 616-715 | 28.08 | 26.39 | 30.28 | 0.74 | 27.57 | 28.49 | -22.33 | -24.77 | -20.83 | 0.64 | -22.65 | -21.93 |
| 716-815 | 27.73 | 25.78 | 29.35 | 0.62 | 27.34 | 28.17 | -22.37 | -23.13 | -21.00 | 0.43 | -22.67 | -22.16 |
| 816-915 | 27.67 | 25.83 | 29.14 | 0.65 | 27.35 | 28.04 | -22.23 | -23.51 | -20.61 | 0.56 | -22.59 | -21.85 |
| 916-1015 | 26.95 | 24.27 | 28.77 | 1.01 | 26.28 | 27.61 | -21.79 | -22.89 | -19.86 | 0.51 | -22.09 | -21.59 |
| 1016-1115 | 26.82 | 24.74 | 28.96 | 0.85 | 26.32 | 27.41 | -21.48 | -22.60 | -20.56 | 0.41 | -21.75 | -21.22 |
| 1116-1215 | 27.11 | 25.02 | 29.33 | 0.87 | 26.44 | 27.86 | -22.15 | -23.97 | -20.55 | 0.78 | -22.68 | -21.43 |
| 1216-1315 | 27.34 | 25.22 | 31.90 | 1.00 | 26.85 | 27.89 | -21.85 | -23.75 | -20.45 | 0.74 | -22.43 | -21.27 |
| 1316-1415 | 27.18 | 27.73 | 29.15 | 0.60 | 26.87 | 27.53 | -22.33 | -23.79 | -20.53 | 0.66 | -22.73 | -21.87 |
| 1416-1515 | 26.63 | 24.85 | 28.76 | 0.79 | 26.14 | 27.12 | -22.50 | -24.43 | -20.54 | 0.85 | -22.99 | -21.95 |
| 1516-1615 | 26.35 | 23.85 | 28.98 | 1.14 | 25.52 | 27.12 | -22.24 | -23.30 | -20.63 | 0.62 | -22.72 | -21.78 |
| 1616-1715 | 26.74 | 24.11 | 29.15 | 0.98 | 26.12 | 27.48 | -22.14 | -22.11 | -24.43 | 0.80 | -22.69 | -21.56 |
| 1716-1815 | 26.85 | 24.54 | 29.18 | 0.89 | 26.29 | 27.41 | -21.86 | -23.44 | -20.36 | 0.66 | -22.30 | -21.43 |
| 1816-1915 | 27.19 | 24.14 | 29.75 | 1.25 | 26.26 | 28.05 | -21.50 | -22.79 | -20.36 | 0.48 | -21.83 | -21.14 |
| 1916-2015 | 27.14 | 23.97 | 32.2 | 1.97 | 25.80 | 27.83 | -21.14 | -23.76 | -19.94 | 0.56 | -21.43 | -20.78 |
| 516-2016 | 27.19 | 19.95 | 32.20 | 1.15 | 26.48 | 27.90 | -22.04 | -24.76 | -19.61 | 0.75 | -22.57 | -21.48 |

**Table S3.** Climatic parameters used for reconstructions

| **Parameter** | **Equation for climate reconstruction** |
| --- | --- |
| Reconstruction of the climate parameter | Rec*_t_*= *a*•I*_t_* +*b*+e*_t_*, where Rec*_t_* – reconstruction of the climate parameter; e*_t_ -* component of temperature variability which is not explained by the variability of the stable isotope variation; I_t_ – δ^13^C_cell_ or δ^18^O_cell_, while *a* and *b* - intercept variables. |
| July precipitation reconstruction derived from δ^13^C_cell_ | P_July_ = (-762.87 + (-38.47 • δ^13^C_cell_)) + 0.14 |
| July air temperature reconstruction derived from δ^18^O_cell_ | T_July_ = (2.62 + (0.41 • δ^18^O_cell_)) + 0.11 |
| Regional June-July-August (JJA) precipitation reconstruction based on δ^13^C_cell_ combined with Co/Inc and Rb/Sr from Teletskoe Lake core sediments (TLs) | Prec_JJA (mm) = -110.72 • Co/Inc -103.78 • Rb/Sr -32.84 • δ^13^C_cell_ – 488.78 |
| Regional June-July-August (JJA) air temperature reconstruction based on δ^18^O_cell,_ TRW, MXD and elemental concentrations (Ca, Ti, Br/Sr) in the Teletskoe Lake core sediments (TLs) | ASMR-JJA (°C) = 1.13 • Ca – 1.56• Ti+2.43• Br/Sr + 0.53 •TRW+ 0.40 •MXD +0.13 +18.91• δ^18^O_cell_ |

**Table S4.** Statistical characteristics (R- correlation coefficient, R^2^ – determination coefficient, F-criteria – Fischer criteria, DW – Durbin-Watson statistics, К_s_ – coefficient of synchronicity, CE –covariance error, RE- reduction error) for calibration and verification periods for July precipitation from Mugur-Aksy weather station and the δ^13^C_cell_.

| **Calibration** | | | | | **Verification** | | | | | |
| --- | --- | --- | --- | --- | --- | --- | --- | --- | --- | --- |
| Period | R | R^2^ | F-criteria | DW-  statistic | Period | R | R^2^ | К_s_ | CE | RE |
| 1966-2016 | -0.58 | 0.34 | F=18.11  df=1.36  P<0.001 | 1.84 |  |  |  |  |  |  |
| 1966-1990 | -0.38 | 0.15 | F=4.85  df=1.28  P<0.001 | 1.75 | 1991-2016 | 0.48 | 0.23 | 0.53 | 0.2 | 0.1 |
| 1991-2016 | -0.50 | 0.25 | F=9.03  df=1.27  P<0.001 | 1.93 | 1966-1990 | 0.68 | 0.46 | 0.57 | 0.4 | 0.3 |

**Table S5.** Statistical characteristics (R- correlation coefficient, R^2^ – determination coefficient, F-criteria – Fischer criteria, DW – Durbin-Watson statistics, К_s_ – coefficient of synchronicity, CE –covariance error, RE- reduction error) for calibration and verification periods for July air temperature from the Mugur-Aksy weather station and δ^18^O_cell_.

| **Calibration** | | | | | **Verification** | | | | | |
| --- | --- | --- | --- | --- | --- | --- | --- | --- | --- | --- |
| Period | R | R^2^ | F- criteria | DW-  statistic | Period | R | R^2^ | К_с_ | CE | RE |
| 1963-2015 | 0.64 | 0.40 | 33.84  df=1.51  p<0.001 | 1.79 |  |  |  |  |  |  |
| 1963-1989 | 0.40 | 0.16 | 4.85  df=1.25  p<0.001 | 1.72 | 1989-2015 | 0.43 | 0.18 | 0.44 | 0.20 | 0.1 |
| 1989-2015 | 0.43 | 0.18 | 5.70  df=1.25  p<0.001 | 2.25 | 1963-1989 | 0.40 | 0.16 | 0.40 | 0.4 | 0.2 |

**Table S6.** Extreme anomalies (≥ 2.5σ) calculated relative to the whole studied period (516-2016 CE) for July precipitation derived from δ^13^C in larch tree-ring cellulose (δ^13^C_cell_) and July air temperature reconstruction derived from δ^18^O in larch tree-ring cellulose (δ^18^O_cell_).

| **Reconstruction** | **Cold** | | **Warm** | | **Dry** | | **Wet** | |
| --- | --- | --- | --- | --- | --- | --- | --- | --- |
|  | Year | σ | Year | σ | Year | σ | Year | σ |
| **δ^13^C_cell_**  July precipitation reconstruction  (516-2016 CE) |  |  |  |  | 985  1963  1974  2009  2015  2016 | -2.9  -2.8  -2.6  -2.6  -2.7  -2.9 | 686  1487  1489  1706 | 3.6  3.2  3.1  3.2 |
| **δ^18^O_cell_**  July temperature reconstruction  (516-2016 CE) | 536  932  1558  1579  1587  1590  1649  1912  1941  1959  1960  1984 | -6.3  -2.5  -2.9  -2.8  -2.5  -2.8  -2.7  -2.6  -2.8  -2.5  -2.7  -2.8 | 522  526  522  526  601  617  1264  1266  2000  2001  2002  2003  2005  2006  2007  2008  2009  2010  2011  2012  2013  2014  2015 | +3.0  +2.9  +3.0  +2.9  +2.5  +2.7  +2.6  +4.1  +2.7  +4.0  +3.7  +2.8  +3.1  +2.8  +3.0  +4.4  +4.3  +2.8  +2.8  +3.7  +3.2  +2.9  +3.2 |  |  |  | |

**Table S7.** Mean, standard deviation – SD, minimum and maximum values calculated for air temperature and precipitation reconstructions derived from: δ^13^C and δ^18^O in tree-ring cellulose (δ^13^C_cell_ and δ^18^O_cell_); tree-ring width (TRW); maximum latewood density (MXD); ratio of geochemical elements Rb/Sr of the Teletskoe Lake sediments (TLs) and Co/Inc; δ^18^O in ice core (δ^18^O_ice_). Statistical characteristics were calculated for the second part of the first millennium (516-800 CE), Medieval Warm Period (MWP, 800-1300 CE), Little Ice Age (LIA, 1400-1900 CE) and recent period (RP, 1900-2000 and 2000-2016 CE).

| **Parameter** | 516-800 CE | | | | 800-1300 CE | | | | 1400-1900 CE | | | | 1900-2000 CE | | | | 2000-2016 CE | | | |
| --- | --- | --- | --- | --- | --- | --- | --- | --- | --- | --- | --- | --- | --- | --- | --- | --- | --- | --- | --- | --- |
|  | Mean | SD | Min | Max | Mean | SD | Min | Max | Mean | SD | Min | Max | Mean | SD | Min | Max | Mean | SD | Min | Max |
| **δ^13^C_cel_**  July precipitation, (mm) | 102.3 | 20.6 | 38.7 | 190.1 | 79.6 | 25.6 | 1.4 | 159.4 | 86.2 | 29.6 | 20.4 | 177.3 | 54.3 | 20.6 | 4.6 | 151.2 | 32.4 | 21.7 | 0.1 | 88.3 |
| **δ^18^O_cell_**  July air temperature, (°C) | 14.2 | 0.4 | 10.9 | 15.3 | 13.9 | 0.4 | 12.7 | 15.8 | 13.7 | 0.4 | 12.5 | 14.9 | 13.6 | 0.5 | 12.6 | 15.1 | 15.3 | 0.3 | 14.8 | 15.9 |
| **TRW**  June-July air temperature,  (°C) | 7.9 | 0.41 | 6.9 | 9.1 | 8.1 | 0.5 | 6.7 | 10.0 | 8.1 | 0.6 | 6.7 | 10.1 | 8.9 | 0.6 | 6.9 | 9.9 | 8.9 | 0.8 | 7.7 | 10.6 |
| **MXD**  June-July-August air temperature  **(z-score)** | -0.4 | 0.8 | -2.8 | 1.7 | -0.3 | 0.9 | -2.9 | 2.7 | 0.3 | 0.9 | -2.3 | 3.2 | 0.7 | 0.7 | -1.5 | 2.1 | 1.1 | 0.6 | -0.2 | 1.9 |
| **Co/Inc**  July precipitation  Lake sediments,  **(z-score)** | 0.6 | 0.1 | 0.4 | 0.7 | 0.6 | 0.1 | 0.4 | 0.7 | 0.6 | 0.1 | 0.4 | 0.7 | 0.5 | 0.1 | 0.4 | 0.6 | NA | NA | NA | NA |
| **Rb/Sr**  June-July-August air temperature  Lake sediments,  **(z-score)** | 0.7 | 0.6 | 0.3 | 1.1 | 0.6 | 0.1 | 0.3 | 0.9 | 0.6 | 0.1 | 0.2 | 1.1 | 0.6 | 0.1 | 0.5 | 0.7 | NA | NA | NA | NA |
| **δ^18^O_ice_**  March-November air temperature,  **(z-score)** | NA | NA | NA | NA | -0.0 | 0.5 | -0.9 | 0.5 | -0.2 | 1.0 | -3.0 | 2.2 | 1.6 | 0.7 | 0.9 | 2.7 | NA | NA | NA | NA |
| **ASMR summer air temperature,**  **(z-score)**  (δ^18^Ocell+ TLs Ca, TLs Ti+ TLs Br/Sr + TRW+ MXD) | -0.3 | 1.00 | -2.4 | 3.6 | 0.0 | 0.9 | -2.5 | 3.2 | -0.0 | 1.0 | -3.0 | 3.8 | 0.7 | 1.0 | -2.4 | 2.6 | 1.9 | 1.0 | 0.7 | NA |
| **ASMR July precipitation,**  **(mm)**  (δ^13^Ccell+ TLs Rb/Sr, TLs Co/Inc) | 116.1 | 13.4 | 80.6 | 147.8 | 103.3 | 20.2 | 58.1 | 144.9 | 106.7 | 26.5 | 43.8 | 168.9 | 84.7 | 6.3 | 74.6 | 100.5 | 85.8 | 2.9 | 79.4 | NA |
| **PAGES 2K,**  **(z-score)** | -0.6 | 0.1 | -1.0 | -0.3 | -0.6 | 0.1 | -0.8 | -0.4 | -0.7 | 0.1 | -1.0 | -0.4 | -0.3 | 0.2 | -0.7 | 0.1 | -0.6 | 0.1 | -1.0 | NA |

Note: *1900-2016 for July air temperature and July precipitation reconstructions derived from δ^18^O_cell_ and δ^13^C_cell_, respectively.

NA- not available
